# Decoding e-waste challenges with hybrid AHP and ISM Model approach: An initiative towards a cleaner future in India

**DOI:** 10.12688/f1000research.167041.1

**Published:** 2025-09-02

**Authors:** Ashok Kumar Singhdeo, Sushanta Tripathy, Deepak Singhal

**Affiliations:** 1 School of Mechanical Engineering, Kalinga Institute of Industrial Technology, Bhubaneswar, Odisha, 751024, India

**Keywords:** e waste, e-waste mitigation, challenges, ISM, AHP, sustainability.

## Abstract

**Background:**

E-waste has rapidly become the fastest growing waste stream in the world, posing serious threats to environmental sustainability and the global agenda for a cleaner, greener, and more prosperous future. Countries that are relatively new to e-waste management as India, face significant challenges in identifying and addressing the barriers to effective mitigation efforts.

**Methods:**

This study employs the Interpretive Structural Modelling (ISM) technique to decode and structure six levels of barriers leading to the seventh and most critical barrier: lack of sustainable design practices. In addition, a MICMAC analysis is used to classify the identified e-waste challenges as either driving or dependent variables, offering strategic insights for stakeholders. To further validate the findings, the Analytic Hierarchy Process (AHP), a multi-criteria decision-making (MCDM) method, is applied to assign weightage to each challenge based on expert input and to assess the robustness of the data.

**Results:**

The analysis reveals the absence of autonomous factors in the e-waste challenge ecosystem. Key dependent factors include fluctuating supply chains, underdeveloped R&D practices, lack of safety protocols, and poor integration of sustainable design practices. These are influenced by key driving constraints such as illegal e-waste practices, inconsistent policies and regulations, poor coordination, and limited accessibility. These driving challenges act as precursors to the ultimate barrier: the lack of sustainable design practices.

**Conclusions:**

The study provides actionable insights and prioritization of challenges that can guide policymakers and business leaders in developing effective e-waste mitigation strategies. The structured model offers a foundational framework for countries—whether newly engaged or planning to implement e-waste management to adopt it as a national agenda for achieving sustainable development goals.

## 1. Introduction

In modern times, e-waste is the “fastest-growing waste stream in the world” with 62 billion Kg generated in 2022 with only 22.3% being recycled raising concerns and aptly quoted by UN (2022) as “tsunami of e-waste”. Its estimated value is $91 billion. Since 2021 and 2022 saw 3.4% global electronic waste rise, the world is on alert. “6R” and the “circular economy,” emerging paradigms, could boost global commercial activity ( Ahmad et al., 2024).

In 1970, with the establishment of Environmental Protection Agency [EPA); USA], a responsible approach began for creating standards and laws to promote health of individuals and environment ( Pisano, 1976; Brook et al., 2024). In 1976 ( Ali et al., 2024), the Resource Conservation and Recovery Act (RCRA), formed as a law with an objective to ensure the management of waste in an environment-friendly way. Basel convention (1989), an international treaty designed to reduce movements of hazardous waste between nations from developed to developing countries ( Montgomery, 1990; Chen et al., 2024). These two e-waste act and treaty formed the basis for propelling of the e-waste recycling industry in the developed countries. As of October 2019, 78 countries globally have established a policy, legislation or specific regulation to govern e-waste. Table 1 depicts the significant steps of the evolution of e-waste regulations/initiatives around the globe.

**Table 1.** Evolution of e-waste regulations globally.

YEAR	COUNTRY	DESCRIPTION
1976	USA	Resource Conservation and Recovery Act (RCRA) passed
1991	Switzerland	Refrigerators collected as e waste recovery
2001	European Union	Collection of electrical and electronic equipment
2004	Canada	Electronics recycling fee introduced
2006	Brazil	National policy for e-waste management
2008	China	• Draft regulation on the management of electronic waste • Ban on import of e-waste
2008	South Africa	Established eWASA; an ecofriendly e waste management system
2008	East Asia [South Korea, Japan, Taiwan]	Ensured manufacturers responsibility to recycle 75% of e-waste of their annual production
2010	Australia	National Waste Policy endorsed.
2012	India	e-waste (Management and Handling) Rules implemented
2013	Chile	• Established a framework for waste management • Encouraged recycling of e-waste
2013	Columbia	EPR (Extended Producer Responsibility) law introduced to manage e-waste
2014	Israel	National e-waste law introduced
2020	New Zealand	e-waste: a priority product in Waste Minimization Act (2008)

Although, e-waste is quantity and control specific and nation-specific, approaches towards e-waste mitigation are formidable in their own contexts, an overall approach to e-waste management challenges in developing countries is yet to be captured. This research presents a fresh view of e-waste implementation barriers and their prioritization along with a model to decode the path of mitigation approach in Indian context. It can also be viewed as trends in developing economies of the world in upcoming e-waste industry. To implement mitigation strategies, the challenges are to be identified and ranking them with weightage and build a model to showcase the direction of attacking these challenges is the need-of-the-hour to resolve the issues. The lack of an integrated analysis of e-waste barriers in e-waste industry context of India intrigued the authors to begin the research to identify the barriers in e-waste implementation. Further, the challenges are prioritized through ranking and hence a clear picture of understanding evolves for the stakeholders. This led the authors to propose the research questions (RQs) for this research:

***RQ1:** To **identify** the barriers in e-waste implementation in Indian context (?)*

***RQ2: To prioritize/rank** the challenges identified through weightage to decode the importance of each of the challenges (?)*

***RQ3:** To **build a model** to trace the focus barriers for implementation of e-waste initiatives (?)*

***RQ4:** To **suggest insights for strategy** to mitigate the e-waste challenges (?)*

**RQ1** was addressed through the vast literature survey, experts’ opinion while the formidable MCDM methods viz. Analytic Hierarchy Process (AHP) and Interpretive Structural Modeling (ISM) were used to rank the challenges through weightage and build the model ( **RQ2** and **RQ3**). **RQ4** is the novelty of the research where the authors propose insights which would act as the base for mitigation strategies to alleviate the barriers in e-waste management initiatives.

**Figure 1.**
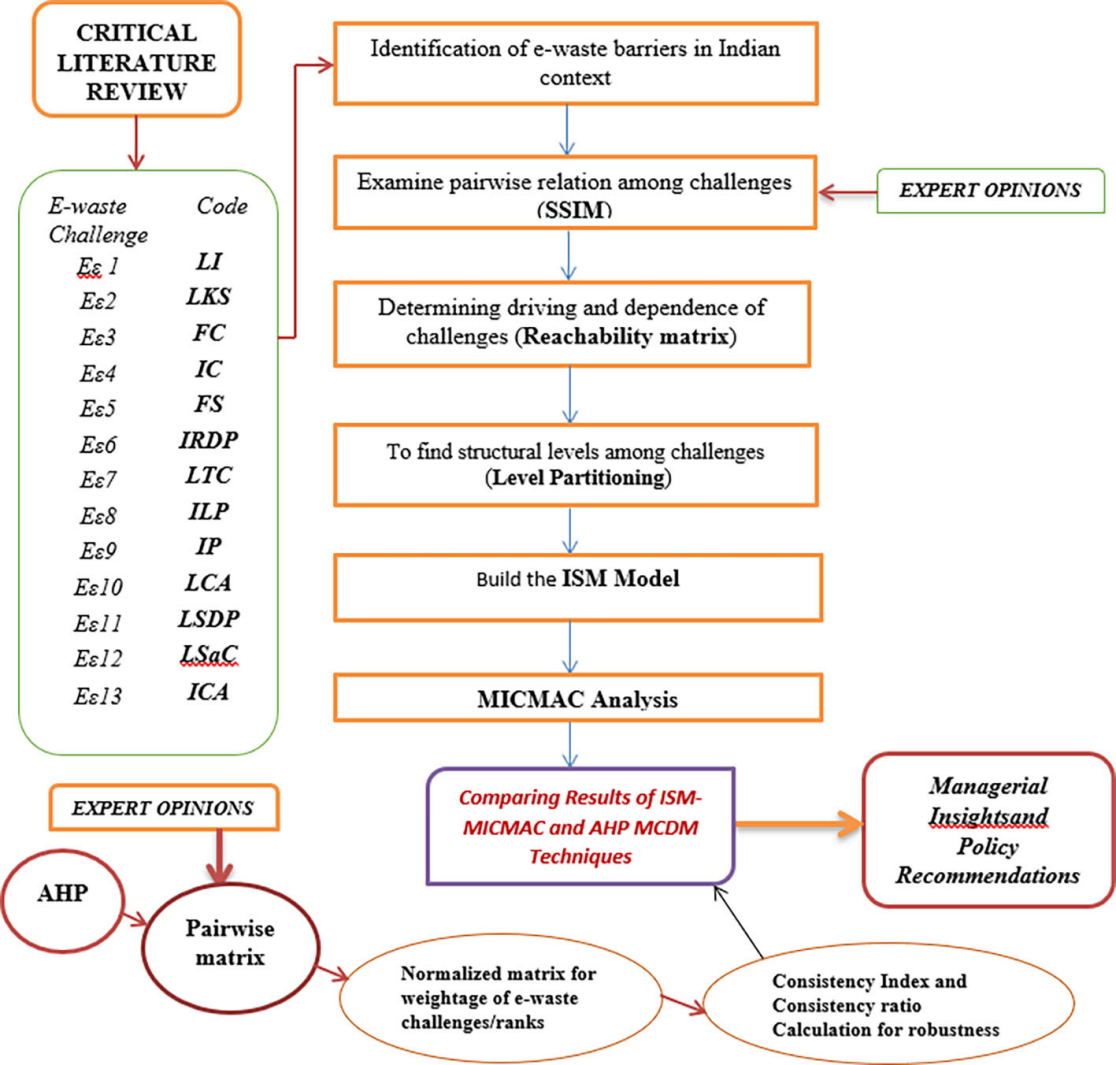
Framework of e-waste challenge research.

The framework of the paper is as follows: Section 2 showcases the literature pertaining to e-waste barriers resulting in Table 2; Section 3 delves into the research design/methodology section which showcases the design framework in Figure 1 and describes two MCDM techniques viz. ISM and AHP; Section 4 involves the results; Section 5 comprises the discussion of findings; Section 6 showcases the managerial implications; Section 7 concludes the research.

**Table 2.** Literature survey for identification of e-waste challenges with codes.

Hindrance (Εε) no.	Author	Focus	e-waste challenge identified	Code
*Εε1*	*Bagwan (2024)* *Shahabuddin et al. (2023)* *Murthy and Ramakrishna (2022)*	*Infrastructural capacity building for e waste recycling*	*Lack of Infrastructure*	*LI*
*Εε2*	*Sundar et al. (2023)* *Yadav et al. (2022)* *Kwatra et al. (2014)*	*Knowledge and skill aspects*	*Lack of Knowledge and Skills*	*LKS*
*Εε3*	*Awino and Apitz (2024)* *Alblooshi et al. (2022)* *Ofori and Opoku Mensah (2022)*	*Financial constraints in waste hierarchy and circular economy frameworks*	*Financial Constraints*	*FC*
*Εε4*	*Mohideen et al. (2024)* *Dutta et al. (2023)* *Almulhim (2022)*	*Lack of information and low awareness levels*	*Information Constraints*	*IC*
*Εε5*	*He et al. (2024)* *Joshi et al. (2023)* *Wang et al. (2022)* *Sengupta et al. (2022)*	*Supply chain hindrances in e waste implementation for sustainable circular economy*	*Fluctuating Supply*	*FS*
*Εε6*	*Neves et al. (2024)* *Ilankoon et al. (2024)* *Ni et al. (2023)* *Hansen et al. (2022)*	*Promoting increase of e waste recycling; strategies and challenges*	*Immature R&D Practices*	*IRDP*
*Εε7*	*Mallick et al. (2023)* *Asibey et al. (2022)* *Li et al. (2022)* *Lara et al. (2019)*	*Logistics and Transportation issues*	*Logistics & Transportation practices*	*LTC*
*Εε8*	*Faibil et al. (2023)* *Rajesh et al. (2022)* *Thakur and Kumar (2022)* *Srivastava and Pathak (2020)* *Rahman (2017)*	*Inadequate policy and legal framework*	*In Coherent Legal and Policy constraints*	*ILP*
*Εε9*	*Rauf (2024)* *Liu et al. (2023)* *Andeobu et al. (2023)* *Santos and Ogunseitan (2022)*	*Mismanagement of e-waste in developing nations*	*Illegal practices*	*IP*
*Εε10*	*Gaur et al. (2024)* *Thukral et al. (2023)* *Sabbir et al. (2023)*	*Customer attitude focus in e waste implementation*	*Lack of customer’s attitude*	*LCA*
*Εε11*	*Nandy et al. (2022)* *Zhang et al. (2022)* *Xu et al. (2020)*	*Improvement strategies to sustainable practices*	*Lack of Sustainable Design Practices*	*LSDP*
*Εε12*	*Bimpong et al. (2024)* *Akbar et al. (2024)* *Roy et al. (2022)*	*Safety regulations in e-waste sector*	*Lack of Safety constraints*	*LSaC*
*Εε13*	*Leclerc and Badami (2024)* *Maes and Whyte (2022)* *Jain et al. (2022)*	*Empirical investigation into municipalities’ contributions to and perspectives on e **-**waste management*	*Improper Coordination and accessibility*	*ICA*

## 2. Literature review

The authors conceiving this research have delved deep into literature pertaining to challenges in e-waste management around the globe through databases viz., Scopus, Web of Science, and Google Scholar. While identifying the challenges they typically explored the varied country wise dispersion of e-waste mechanisms in different scales. These challenges were observed to be generic to most nations globally, especially developing ones. The authors employed latest selection criteria ( van Wee and Banister, 2023) to find the relevant articles. Keywords such as challenges, hindrances, barriers, hurdles, critical success factors were used in the context of e-waste barriers mitigation in varied e-waste demography worldwide.

A total of 185 research papers initiated the search followed by a four-tier screening process for article exclusion involving screening parameters like title (63), abstract (42), removal of duplicates (10), and full-text (25) leading to a final 45 latest research articles inclusion for the review process.13 challenges ( Table 2) for e-waste management got decoded in the process which would suffice the present research objectives.

The literature that follows highlights the awareness, global recent trends, and existing Indian approach to e-waste management issues till-date.

Generally defined as “discarded electrical or electronic devices, its components or equipments,” electronic trash, sometimes known as e-waste, also referred to as waste electrical and electronic equipment (WEEE) or end-of-life (EOL) electronics ( Baldé et al., 2017). The growing consumption of e-goods as a part of digital revolution and science and technology innovations has led to this gigantic magnitude of global e-waste hazard ( Perkins et al., 2014). As an illustration, 70 % of toxic waste in landfills is contributed by e-wastes. As a trend, 6R and circular economy tool/concept are applied to alleviate e-waste chaos to some extent. The United States Environmental Protection Agency (USEPA) classifies e-waste into ten categories viz., large household appliances, small household appliances, IT equipment, consumer electronics, lamps and luminaries, toys, tools, medical devices, monitoring and control instruments, and automatic dispensers. The Guiyu, China is the “e-waste capital of the world” ( Wong et al., 2007) as it recycles maximum proportions of traditional e-wastes by transforming the city to an “e-waste recycling centre”.

In the last decade, with digital revolution, many digitized products such as cryptocurrency has added to the woes due to its global reach with lightning speed in the entire globe. Bitcoin mining, being one of them, is used for payment or speculative purposes have contributed enormously to e-waste generation. De Vries and Stoll (2021) reported in their research that an average bitcoin transaction yields 272 grams of electronic waste which approximated to 112.5 million grams of waste in the year 2020. The bitcoin waste disposal rate exceeds that of major financial organizations such as VISA ( Jana et al., 2021). The bitcoin industry’s rapid turnover of technology leads to higher levels of e-waste. Using the ‘proof-of-work’ principle, the miners are encouraged towards competitiveness through a reward system which drives them to acquire latest e-products thereby increasing the volume of discarded e-products. Miners are also incentivized to purchase new chips to gain competitive advantage in cryptocurrency domains governed by the Kroomy’s law i.e. “efficiency of computer chips doubles every 1.5 years”. The discarded chips add-up as e-wastes due to their non-reusable and non-refurbishable limitations. Much of bitcoin waste end up in landfills along with 83.6% of the total e-wastes ( Saleh, 2021). Some studies suggest bitcoin network reduces small IT and Telecommunication equipment (SITTE) waste thereby impacting positively in countries like Netherlands.

India is the third largest producer of e-waste globally. According to ASSOCHAM, an industrial body in the country, the compound annual growth rate of electronic waste is 30%. 65 Indian cities generate more than 60% of India’s total e-waste, the top five being Mumbai, Delhi, Bengaluru, Chennai, and Kolkata respectively. The United Nations Trade and Development (UNCTAD) in its report for 2022, presents a critical statistic as India’s growth rate in generating e-waste as components, devices, and equipment on a global scale is at a colossal 163%. Further, The Digital Economy report (2024) under the theme “Shaping an environmentally sustainable and inclusive digital future” showcases the nation doubling its shares in Small IT and Telecommunication equipment (SCSIT) waste generation in the world i.e. 3.1% [2010] to 6.4% [2022]. Further, the disposal techniques of e-wastes in India is largely informal, gross, and unstructured, unprotected by any by any enforcement laws and regulations raising contamination of environment (air, water, and soil) leading to health issues thereby impacting social and environmental sustainability directly ( Joon et al., 2017). Due to massive dominance of informal sector and non-existent formal e-waste disposal facilities, the government has a massive challenge to address for achieving the goals of sustainability ( Krikke, 2008). Table 2 showcases the authors, their contribution/focus of research, e-waste challenges decoded, and their codes to be used in this research.

### 2.1 e-waste challenges identified from literature

13 key challenges were identified that are a hindrance to e-waste alleviation through management in India. A brief overview of the various challenges that pose a threat to e-waste mitigation and their current status is as follows:

*Εε1: Lack of Infrastructure* **[LI]**

The e-waste management practices used in developed countries cannot be implemented in India due to unavailability of adequate number of industries to address the initiation of the mitigation process through 6R and circular economy implementation. Thus, the governance, investors and all stakeholders need to work on a vision to capacitate India to unlock its infrastructural prowess. Presently, it is a hindrance to be dealt with firmly ( Bagwan, 2024).

*Εε2: Lack of Knowledge and Skills* **[LKS]**

The recent decade brought the concept of e-waste mitigation to India. As obvious, it got cladded with a fundamental issue of exposure to the novel problem and approach to solve them. No knowledge and skills or means to acquire them is still a critical root-level hindrance to initiate the efforts towards the success of this hazard alleviation ( Sundar et al., 2023).

*Εε3: Financial Constraints* **[FC]**

The cost aspects of introducing any new idea such as e-waste mitigation has its own problems and constraints amidst the potential catastrophe it possess globally if not addressed with diligence. This constraint of finance is due to lack of motivation of investors, government support initiatives and diversion of funds to fuel this mission on an urgent basis. Hence, it becomes a basic challenge to be eradicated for the success of this mitigation initiative ( Awino and Apitz, 2024).

*Εε4: Information Constraints* **[IC]**

Lack of public awareness and data accessibility issues due to lack of transparency or security concerns escalate the problems of resolving the e-waste barriers and is a result of the cumulative effect of basic barriers identified. It aggravates further into low customer awareness which triggers the collapse of the entire endeavor. Hence, it is a vital challenge to be addressed to initiate the mitigation and transformation process towards sustainability ( Mohideen et al., 2024).

*Εε5: Fluctuating Supply* **[FS]**

Constraints like lack of information and customers’ attitude leads to an unstable and fluctuating supply input to the production process of e-waste, which eventually increases costs and hence in combination aggravates the challenge of e-waste mitigation in India ( Debnath et al., 2023). It is an enormous barrier for sustenance of infrastructural facilities to initialize the mitigation mission ( He et al., 2024).

*Εε6: Immature R&D Practices* **[IRDP]**

India is only over a decade young into e-waste mitigation management issues. The know-how to initiate any new thought system has not availed adequate timeframe leading to immature R&D practices with execution flaws leading to a hindrance in achieving the e-waste sustainability goal ( Neves et al., 2024).

*Εε7: Logistics & Transportation practices* **[LTC]**

Logistics and transportation being the backbone of any supply chain, in mitigating e-waste it has a vital role to play. In this context, the lack of industries incorporating 6R and inadequate demand and supply act as precursors to increase costs of operation, hence, enhancing the hurdles towards mitigating e-wastes in India ( Mallick et al., 2023).

*Εε8: Incoherent Legal and Policy constraints* **[ILP]**

In India, laws do exist for e-waste alleviation but is redundant as availability of suitable industries or big companies imbibing circular economy is sparse. Further, appropriate policies can’t be defined without a threshold ambience of certain constraints’ alleviation as precursors. This trade-off is a serious concern to be addressed ( Faibil et al., 2023).

*Εε9: Illegal practices* **[IP]**

As the dominating informal and unorganized sector operates in India for e-waste recycling in a very gross way, to maximize profits they often retort to illegal practices such as import and dumping, and unethical approaches to recycle that enhance toxicity of the environment thereby hindering the core goal of achieving e-waste sustainability ( Rauf, 2024).

*Εε10: Lack of customer’s attitude* **[LCA]**

The most vital precursor challenge in e-waste transformation into sustainable paradigms in the customer-driven market is their attitude towards 6R products which include taboos, social status quo issues, psychological resistance due to older ideals etc. It is one of the most powerful barriers to be overcome for initiation mechanism of e-waste mitigation ( Gaur et al., 2024).

*Εε11: Lack of Sustainable Design Practices* **[LSDP]**

Lack of sustainable practices act as a hindrance as an overall sum of all the other 12 challenges identified in the literature. Design is a combination of numerous sustainable aspects and a right balance blending is very difficult to achieve. Under these initial years of Indian struggles to alleviate e-waste challenges, it is a goal of colossal magnitude to be achieved ( Nandy et al., 2022).

*Εε12: Lack of Safety constraints* **[LSaC]**

Constraints like lack of knowledge and skills, finance, and awareness of workforce cumulatively add-up to safety issues through various aspects such as non-mastery in work, unaffordability to provide mechanisms and measures, and human errors respectively. The first two of these aspects are constraint-driven and can be addressed towards minimization which is yet to be achieved. This hinders and demotivates the workforce as risk increases, hence, a vital challenge to e-waste mitigation prospects ( Bimpong et al., 2024).

*Εε13: Improper Coordination & accessibility* **[ICA]**

Coordination of unstructured workforce and their accessibility to various resources such as skills and knowledge levels, and information brings forth a hazy picture of their work prospects psychologically leading to demotivation and brooding conflicts. It leads to failure of the goal of sustainable e-waste management achievements and hence an important challenge to be conquered ( Leclerc and Badami, 2024).

## 3. Research design

To fulfill the objectives of this research, the authors have employed a proven and formidable design. The design involves a sequence of steps as depicted in the framework ( Figure 1) which involves two robust MCDM Techniques viz., Interpretive Structural Modeling (ISM) and Analytic Hierarchy Process (AHP). Extensive literature review followed by experts’ validation empowered the authors for data-driven model building followed by MICMAC analysis to categorize the challenges after determining the relationships between them through an ISM model. Analytic hierarchical Process (AHP) was employed to further enhance the robustness and consistency of the model through validation of expert opinion data taken on a 9-point scale. Figure 1 showcases the framework of the research design ( Mohapatra et al., 2022).

### 3.1 Data collection

The authors, through a rigorous literature survey identified 13 hindrances to e-waste management initiation in Indian context. These challenges were to be validated by the experts to identify the significant ones for management approach and discarding the others. Two robust MCDM techniques viz., Analytic Hierarchy Process (AHP) and Interpretive Structural Modeling (ISM) were employed, the former to deduce weightage of challenges along with coherency of experts’ opinions as a validity check while the latter to identify levels of challenges identified which are to be resolved from level 7 (Short-term focus/basic or root barriers) and gradually building to the most complex (Long-term focus/advanced barrier) at level 1 through an ISM model. The need for data collection arose as both these MCDM techniques get initiated through meticulous opinions of experts which they cultivated through their work experiences and observations ( Podvezko, 2009).

For AHP as well as ISM, a total of same 15 experts responded at one point of time, 9 from research/academia and 6 from industries with e-waste management experience. For evaluating preferences in AHP, a 9-point scale was used which reads as: 1: Equal importance; 2: Equal to moderate importance; 3: Moderate importance; 4: Moderate to strong importance; 5: Strong importance; 6: Strong to very strong importance; 7: Very strong importance; 8: Very to extreme strong importance; 9: Extreme importance. 16 challenges identified through literature survey when validated through AHP led to a final 13 barriers qualified for managerial analysis. These are coded as h-1 to h-13 for analysis purposes. The 3 insignificant challenges discarded involve unstable composition (of e-waste products), uneven escalation of e-waste generation, and weak stakeholder communication/collaboration. For ISM model the experts gave their valuable insights through a 5-point Likert scale interpreted as: 1: No importance; 2: Low importance; 3: Importance; 4: High importance; 5: Very High Importance. The number of experts for both AHP and ISM are within appropriate limits. AHP gives better results with greater sample size while for ISM, the number of experts can start from as low as 3. These 13 challenges finalized whose alleviation would mitigate e-waste were to be put into a structure or sequence (levels) through a model, which would help resolve them from basic to advanced challenges.

### 3.2 AHP

Saaty (1990) developed a practical method for decision makers to gain insights to numerous subjective, complicated, and conflicting criteria through a robust MCDM technique, the Analytic Hierarchy Process (AHP). The steps of AHP are elucidated ( Khoshand et al., 2019) in the following sub-sections:

*3.2.1 Pairwise matrix*

The insights of experts collected through the 9-point scale which occupy the upper-triangular portion of the ( *n* x *n)* matrix showcases a hindrances’ relationship with other (n-1) hindrances. The diagonal elements are valued at 1 as they refer to the same hindrance. The lower triangular portion are given reciprocal values of experts’ opinions i.e. if a _12_ = 3, then a _21_ = 1/3 as it is a pairwise influence comparision. 15 experts provided opinions as a matrix whose cell-wise matrix were averaged to find average pairwise matrix. It forms the base for the entire calculations involved in AHP. The column sums of each of the 13 columns were summated.

*3.2.2 Normalized weightage matrix*

Each column sum obtained in pairwise matrix is divided in their respective column in pairwise matrix to obtain the normalized matrix. Then, average of each row of normalized matrix results in weightage of each criterion which is ranked accordingly.

*3.2.3 Model robustness*

The criteria weights are multiplied to each cell of column and row wise in two steps. The first step involves multiplying criteria weights of each hindrance to the entire column of the same hindrance, similarly, for all hindrances in the pairwise matrix ( Table 5). Then, each criteria weight for the hindrance is multiplied row-wise to obtain the final cell values. The weighted sum of each hindrance is obtained by row summation of each hindrance of Table 6. X represents a parameter of ratio between weighted sums for each hindrance to respective criteria weight.

**Table 3.** Pairwise comparison matrix.

Barriers	*Εε1*	*Εε2*	*Εε3*	*Εε4*	*Εε5*	*Εε6*	*Εε7*	*Εε8*	*Εε9*	*Εε10*	*Εε11*	*Εε12*	*Εε13*
*Εε1*	1	2.5	1.5	1.25	1.25	1.5	1.5	1.25	1.5	1.5	1.5	3	2
*Εε2*	0.4	1	2	3.6	3	3.5	2	2.5	3	3.8	4	3	3
*Εε3*	0.67	0.5	1	2	3	2	1.5	2	2	2	4.8	2	3.5
*Εε4*	0.8	0.28	0.5	1	3	2.4	2	3	4	2	4	3	3.5
*Εε5*	0.8	0.33	0.33	0.33	1	1.5	2	2	2	3	2	2	2
*Εε6*	0.67	0.29	0.5	0.42	0.67	1	2	4	6	3	8	3	2
*Εε7*	0.67	0.5	0.67	0.5	0.5	0.5	1	5.2	7.2	2	5	4	3
*Εε8*	0.8	0.4	0.5	0.33	0.5	0.25	0.19	1	7	2	9	3	2
*Εε9*	0.67	0.33	0.5	0.25	0.5	0.17	0.14	0.14	1	2	3	1.25	1.5
*Εε10*	0.67	0.26	0.5	0.5	0.33	0.33	0.13	0.13	0.5	1	3	1.25	1.5
*Εε11*	0.67	0.25	0.21	0.25	0.5	0.13	0.2	0.11	0.33	0.33	1	1.25	1.5
*Εε12*	0.33	0.33	0.5	0.33	0.5	0.33	0.25	0.33	0.8	0.8	0.8	1	1.25
*Εε13*	0.5	0.33	0.29	0.29	0.5	0.5	0.33	0.5	0.67	0.67	0.67	0.8	1
SUM	8.63	7.31	8.99	11.05	15.25	14.11	13.24	22.16	36	24.1	46.77	28.55	27.75

**Table 4.** Normalized weighted matrix.

Barriers	Εε1	Εε2	Εε3	Εε4	Εε5	Εε6	Εε7	Εε8	Εε9	Εε10	Εε11	Εε12	Εε13	Criteria weights	Rank
*Εε1*	0.23	0.31	0.13	0.07	0.05	0.06	0.09	0.04	0.03	0.06	0.02	0.1	0.06	0.1	2
*Εε2*	0.09	0.12	0.17	0.21	0.13	0.14	0.12	0.07	0.06	0.15	0.06	0.1	0.09	0.12	1
*Εε3*	0.15	0.06	0.09	0.12	0.13	0.08	0.09	0.06	0.04	0.08	0.08	0.06	0.1	0.09	3
*Εε4*	0.19	0.03	0.04	0.06	0.13	0.09	0.12	0.09	0.08	0.08	0.06	0.1	0.1	0.09	3
*Εε5*	0.19	0.04	0.03	0.02	0.04	0.06	0.12	0.06	0.04	0.12	0.03	0.06	0.06	0.07	5
*Εε6*	0.15	0.04	0.04	0.02	0.03	0.04	0.12	0.12	0.11	0.12	0.13	0.1	0.06	0.08	4
*Εε7*	0.15	0.06	0.06	0.03	0.02	0.02	0.06	0.16	0.14	0.08	0.08	0.13	0.09	0.08	4
*Εε8*	0.19	0.05	0.04	0.02	0.02	0.01	0.01	0.03	0.13	0.08	0.14	0.1	0.06	0.07	5
*Εε9*	0.15	0.04	0.04	0.01	0.02	0.01	0.01	0	0.02	0.08	0.05	0.04	0.04	0.04	6
*Εε10*	0.15	0.03	0.04	0.03	0.01	0.01	0.01	0	0.01	0.04	0.05	0.04	0.04	0.04	6
*Εε11*	0.15	0.03	0.02	0.01	0.02	0	0.01	0	0.01	0.01	0.02	0.04	0.04	0.03	7
*Εε12*	0.08	0.04	0.04	0.02	0.02	0.01	0.02	0.01	0.02	0.03	0.01	0.03	0.04	0.03	7
*Εε13*	0.12	0.04	0.02	0.02	0.02	0.02	0.02	0.01	0.01	0.03	0.01	0.03	0.03	0.03	7

**Table 5.** Consistency index calculation [A].

Criteria weights	0.1	0.12	0.09	0.09	0.07	0.08	0.08	0.07	0.04	0.04	0.03	0.03	0.03
BARRIERS	*Εε1*	*Εε2*	*Εε3*	*Εε4*	*Εε5*	*Εε6*	*Εε7*	*Εε8*	*Εε9*	*Εε 10*	*Εε 11*	*Εε12*	*Εε 13*
*Εε1*	1	2.5	1.5	1.25	1.25	1.5	1.5	1.25	1.5	1.5	1.5	3	2
*Εε2*	0.24	1	2	3.6	3	3.5	2	2.5	3	3.8	4	3	3
*Εε3*	0.27	0.25	1	2	3	2	1.5	2	2	2	4.8	2	3.5
*Εε4*	0.18	0.28	0.25	1	3	2.4	2	3	4	2	4	3	3.5
*Εε5*	0.2	0.2	0.17	0.33	1	1.5	2	2	2	3	2	2	2
*Εε6*	0.25	0.18	0.14	0.24	0.5	1	2	4	6	3	8	3	2
*Εε7*	0.33	0.5	0.33	0.5	0.5	0.5	1	5.2	7.2	2	5	4	3
*Εε8*	0.33	0.2	0.25	0.33	0.14	0.25	0.19	1	7	2	9	3	2
*Εε9*	0.17	0.16	0.17	0.25	0.13	0.17	0.14	0.14	1	2	3	1.25	1.5
*Εε10*	0.24	0.26	0.5	0.5	0.33	0.33	0.13	0.13	0.17	1	3	1.25	1.5
*Εε11*	0.27	0.25	0.21	0.25	0.14	0.13	0.2	0.11	0.14	0.12	1	1.25	1.5
*Εε12*	0.33	0.33	0.5	0.33	0.5	0.33	0.25	0.33	0.5	0.33	0.5	1	1.25
*Εε13*	0.5	0.33	0.29	0.29	0.5	0.5	0.33	0.5	0.33	0.33	0.25	0.5	1

**Table 6.** Consistency index calculation [B].

C.W.	Barriers	*Εε 1*	*Εε2*	*Εε3*	*Εε4*	*Εε5*	*Εε6*	*Εε7*	*Εε8*	*Εε9*	*Εε 10*	*Εε 11*	*Εε12*	*Εε 13*	Weighted sum value	X=Wtd. Sum/Criteria Wt.
0.1	*Εε1*	0.1	0.3	0.14	0.11	0.09	0.12	0.12	0.09	0.06	0.06	0.05	0.09	0.06	1.39	13.9
0.12	*Εε2*	0.02	0.12	0.18	0.32	0.21	0.28	0.16	0.18	0.12	0.15	0.12	0.09	0.09	2.04	17
0.09	*Εε3*	0.03	0.03	0.09	0.18	0.21	0.16	0.12	0.14	0.08	0.08	0.14	0.06	0.11	1.43	15.89
0.09	*Εε4*	0.02	0.03	0.02	0.09	0.21	0.19	0.16	0.21	0.16	0.08	0.12	0.09	0.11	1.49	16.56
0.07	*Εε5*	0.02	0.02	0.02	0.03	0.07	0.12	0.16	0.14	0.08	0.12	0.06	0.06	0.06	0.96	13.71
0.08	*Εε6*	0.03	0.02	0.01	0.02	0.04	0.08	0.16	0.28	0.24	0.12	0.24	0.09	0.06	1.39	17.38
0.08	*Εε7*	0.03	0.06	0.03	0.05	0.04	0.04	0.08	0.36	0.29	0.08	0.15	0.12	0.09	1.42	17.75
0.07	*Εε8*	0.03	0.02	0.02	0.03	0.01	0.02	0.02	0.07	0.28	0.08	0.27	0.09	0.06	1	14.29
0.04	*Εε9*	0.02	0.02	0.02	0.02	0.01	0.01	0.01	0.01	0.04	0.08	0.09	0.04	0.05	0.42	10.5
0.04	*Εε10*	0.02	0.03	0.05	0.05	0.02	0.03	0.01	0.01	0.01	0.04	0.09	0.04	0.05	0.45	11.25
0.03	*Εε11*	0.03	0.03	0.02	0.02	0.01	0.01	0.02	0.01	0.01	0	0.03	0.04	0.05	0.28	9.33
0.03	*Εε12*	0.03	0.04	0.05	0.03	0.04	0.03	0.02	0.02	0.02	0.01	0.02	0.03	0.04	0.38	12.67
0.03	*Εε13*	0.05	0.04	0.03	0.03	0.04	0.04	0.03	0.04	0.01	0.01	0.01	0.02	0.03	0.38	12.67

This value of X is used to calculate Consistency Index (CI) and Consistency Ratio (CR) through the following formulas:

$$\lambda_{\max}=\left(\sum X\right)/n;\;n=13\;\left(\text{challenges}\right)$$ (1)

$$C.I.=\left(\lambda_{\max}-n\right)/\left(n-1\right)$$ (2)

C.R.=C.I./Random number(n=13);R.N.=1.56 (3)

The general thumb-rule for data consistency in AHP is “ *closer the value of C.R. to zero, greater the data consistency, and, the acceptable limits of consistency for C.R. value is less than 0.10*”.

### 3.3 ISM

Interpretive Structural Modeling (ISM) is a popular and proven MCDM technique with data-driven initiation; the data being experts’ opinions in a particular field of expertise. The views from experts are collected for validation after identifying challenges through vast literature survey. The VAXO scale for influence ( Singhal et al., 2018) is employed to examine a pairwise relation through a self-interaction matrix. The sub-section 3.3.1 to 3.3.5 invokes the steps for ISM model building and analysis purposes as follows:

*3.3.1 SSIM*

15 industry experts associated with waste management provided hawk-eye views which enriched the authors to extrapolate the contextual relationship among the e-waste challenges. The nature of the relationship between two factors (x and y) is established using VAXO conditions where:

V: if x influences y; A: if y influences x; X: if x and y influence each other; O: if x and y have no relation.

The SSIM is a precursor to obtaining the reachability matrix.

*3.3.2 Reachability matrix*

It is an outcome of SSIM being converted into a 0 and 1 binary value matrix where if a factor (x) influences factor (y), then the value is 1, otherwise 0. Under this condition, for a factor set [x to y] or [y to x] influence depicted as [x, y] or [y, x] matrix cells position for each of VAXO are:

V:1and0;A:0and1;X:1and1;O:0and0.

Thus, the binary reachability matrix is obtained in Table 8 in which adding rows gives a “Driving Power” column while adding columns of the matrix gives a row “Dependence Power”. These are plotted in as y and x axes in MICMAC analysis (section 3.3.5).

**Table 7.** Structural self-interaction matrix [ ***SSIM***].

*e-waste LI challenge code*	*LKS*	*FC*	*IC*	*FS*	*IRDP*	*LTC*	*ILP*	*IP*	*LCA*	*LSDP*	*LSaC*	*ICA*
*LI*	*O*	*O*	*V*	*V*	*V*	*O*	*V*	*V*	*V*	*V*	*V*	*V*
*LKS*		*O*	*V*	*V*	*V*	*O*	*V*	*V*	*V*	*V*	*V*	*V*
*FC*			*V*	*V*	*V*	*O*	*V*	*V*	*V*	*V*	*V*	*V*
*IC*				*V*	*V*	*A*	*V*	*V*	*V*	*V*	*V*	*A*
*FS*					*X*	*A*	*A*	*A*	*A*	*V*	*V*	*A*
*IRDP*						*A*	*A*	*A*	*A*	*V*	*V*	*A*
*LTC*							*V*	*V*	*V*	*V*	*V*	*V*
*ILP*								*X*	*X*	*V*	*V*	*A*
*IP*									*X*	*V*	*V*	*A*
*LCA*										*V*	*V*	*A*
*LSDP*											*A*	*A*
*LSaC ICA*												*A*

**Table 8.** Reachability matrix.

*Sl no.*		*Code*	*Εε1*	*Εε2*	*Εε3*	*Εε4*	*Εε5*	*Εε6*	*Εε7*	*Εε8*	*Εε9*	*Εε 10*	*Εε 11*	*Εε 12*	*Εε 13*	*Driving power*
*Εε1*		*LI*	1	0	0	1	1	1	0	1	1	1	1	1	1	10
*Εε2*		*LKS*	0	1	0	1	1	1	0	1	1	1	1	1	1	10
*Εε3*		*FC*	0	0	1	1	1	1	0	1	1	1	1	1	1	10
*Εε4*		*IC*	0	0	0	1	1	1	0	1	1	1	1	1	0	8
*Εε5*		*FS*	0	0	0	0	1	1	0	0	0	0	1	1	0	4
*Εε6*		*IRDP*	0	0	0	0	1	1	0	0	0	0	1	1	0	4
*Εε7*		*LTC*	0	0	0	1	1	1	1	1	1	1	1	1	1	10
*Εε8*		*ILP*	0	0	0	0	1	1	0	1	1	1	1	1	0	7
*Εε9*		*IP*	0	0	0	0	1	1	0	1	1	1	1	1	0	7
*Εε10*		*LCA*	0	0	0	0	1	1	0	1	1	1	1	1	0	7
*Εε11*		*LSDP*	0	0	0	0	0	0	0	0	0	0	1	0	0	1
*Εε12*		*LSaC*	0	0	0	0	0	0	0	0	0	0	1	1	0	2
*Εε13*		*ICA*	0	0	0	1	1	1	0	1	1	1	1	1	1	9
		Dependence Power	1	1	1	6	11	11	1	9	9	9	13	12	5	

*3.3.3 Level partition*

Table 9 represents the level partition of hindrances where values of each cell of reachability matrix (with values 1) in rows and columns are considered for reachability set (R-set) and antecedent set (A-set) respectively. Rows with values 1, represent that the hindrance h _x_ (x = 1, 2 …, 13; each row having one x value) i.e. *Εε*1 for instance, influences how many other hindrances whereas column values with 1 implies how many hindrances get influenced by *Εε*1.

**Table 9.** Level partition.

*Challenge no.*	*Reachability set*	*Antecedent set*	*Intersection set*	*Level*
***Εε1***	*Εε1,Εε4,Εε5,Εε6,Εε8,Εε9,Εε10,Εε11,Εε12,Εε13*	*Εε1*	*Εε1*	SEVEN
***Εε2***	*Εε2,Εε4,Εε5,Εε6,Εε8,Εε9,Εε10,Εε11,Εε12,Εε13*	*Εε2*	*Εε2*	SEVEN
***Εε3***	*Εε3, Εε4,Εε5,Εε6,Εε8,Εε9,Εε10,Εε11,Εε12,Εε13*	*Εε3*	*Εε3*	SEVEN
***Εε4***	*Εε4,Εε5,Εε6,ew8,Εε9,Εε10,Εε11,Εε12*	*Εε1,Εε2, Εε3,Εε4,Εε7,Εε13*	*Εε4*	FIVE
***Εε5***	*Εε5,Εε6, Εε11,Εε12*	*Εε1,Εε2,Εε3,Εε4,Εε5,Εε6,Εε7,Εε8,Εε9,Εε10, Εε13*	*Εε5,Εε6*	THREE
***Εε6***	*Εε5,Εε6, Εε11,Εε12*	*Εε1,Εε2,Εε3,Εε4,Εε5,Εε6,Εε7,Εε8,Εε9,Εε10, Εε13*	*Εε5,Εε6*	THREE
***Εε7***	*Εε4,Εε5,Εε6,Εε7,Εε8,Εε9,Εε10, Εε11,Εε12*	*Εε7*	*Εε7*	SEVEN
***Εε8***	*Εε5,Εε6,Εε8,Εε9,Εε10, Εε11,Εε12*	*Εε1,Εε2,Εε3,Εε7,Εε8,Εε9, Εε10, Εε13*	*Εε8,Εε9,Εε10*	FOUR
***Εε9***	*Εε5,Εε6,Εε8,Εε9,Εε10, Εε11,Εε12*	*Εε1,Εε2,Εε3,Εε7,Εε8,Εε9, Εε10, Εε13*	*Εε8,Εε9, Εε10*	FOUR
***Εε10***	*Εε5,Εε6,Εε8,Εε9,Εε10, Εε11,Εε12*	*Εε1,Εε2,Εε3,Εε7,Εε 8,Εε9, Εε10, Εε13*	*Εε8,Εε9, Εε10*	FOUR
***Εε11***	*Εε11*	*Εε1,Εε2,Εε3,Εε4,Εε5,Εε6,Εε7,Εε8,Εε9,Εε10,Εε11,Εε12,Εε13*	*Εε11*	ONE
***Εε12***	*Εε11,Εε12*	*Εε1,Εε2,Εε3,Εε4,Εε5,Εε6,Εε7,Εε8,Εε9,Εε10,,Εε12,Εε13*	*Εε12*	TWO
***Εε13***	*Εε4,Εε5,Εε6,Εε8,Εε9,Εε10,,Εε12*	*Εε1,Εε2,Εε3, Εε7, Εε13*	*Εε13*	SIX

Further, an Intersection set (I-set) is deduced which showcases the common challenges in R-set and A-set.

The procedure for level indexing after the R, A, and I-sets elements identification is encapsulated as:

• *Level 1 (long term focus hindrance) is decided when R-set and I-set have same value. It is placed at the top of the model.*
• *With level 1 identified, we eliminate it from all factors in all three R, A, and I- sets and again check for same R and I-set same “hindrance number” and obtain level 2.*
• *Similarly, we reach till level 7 in the above stepwise manner; levels labeled as L-1 to L-7.*

*3.3.4 ISM model*

The ISM model which uses a diagraph, showing hindrances as nodes or vertices and joined by edges, exhibits interdependence between them. The basic assumption from an ISM model is transitivity i.e. if *Εε*1 influences Εε2, and Εε2 influences Εε3 implies *Εε*1 will influence *Εε3* transitively. The authors have used a Floyd-Marshall algorithm using Python 3.0 code with initial reachability matrix as input to remove transitivity and give final reachability matrix without transitivity as output which is used to generate the model. The model illuminates the structural relationship among the factors. Thus, ISM converts a complex set of numerous factors to a well-established interrelationship for clarity of the research context as a model ( Chakraborty et al., 2023). It represents a clear view of the issue by focusing on the head-on and ancillary relationship between the challenges. It combines the experts’ opinions and their experiential depths in a superior organized way.

*3.3.5 MICMAC analysis*

The driving power and dependence power of each challenge from reachability matrix are plotted in a quadrant chart where the origin is average of 0 and highest dependence power i.e. 13 in x-axis and in y-axis it is average of 0 and 10 dependence power implying origin to be at (6.5,5). His plot is referred to as MICMAC (Matriced’ Impacts croises-multiplication appliqu’e an un Classement) analysis.

Figure 1 shows the framework of e-waste management implementation hindrance research design procedures.

## 4. Results

The results for both AHP and ISM have been determined through calculations using sub-sections 3.2 and 3.3, which are shown as follows:

### 4.1 AHP calculations

The AHP matrix calculations to final weightage and ranks of challenges to e-waste management implementation are represented from Table 3 to Table 6.

### 4.2 ISM calculations

The Tables from 7 to 9 showcase ISM matrix calculations leading to level partition table for model building.

## 5. Discussion

This project intends to look at and establish a structural link among the key components influencing e-waste management. Understanding these obstacles will help us to apply e-waste management and help to lower this global issue. We shall apply the ISM framework for this purpose. Key elements are identified by their driving forces and dependence on MICMAC analysis, therefore providing insightful information. The critical outcomes of this research are discussed in sub-sections 5.1 and 5.2 to showcase the results and observe the trends towards fulfilling the research objectives.

### 5.1 AHP: Hindrance weightage and data validation observations

The second research objective (RQ2) to prioritize or rank the e-waste challenges through their weightage calculations are accomplished by AHP.

The weightage of hindrances to e-waste implementation are ranked as:

Εε2(0.12)>Εε1(0.10)>Εε3=Εε4(0.09)>Εε6=Εε7(0.08)>Εε5(0.07)>Εε9=Εε10(0.04)>Εε11=Εε12=Εε13(0.03)

It shows the importance of various challenges to e-waste implementation.

Lack of knowledge and skills tops the weightage with 12% followed by lack of infrastructure (10%). Financial and information constraints are equally important with 9% weightage each, placed in third position. The fourth position is a tie between immature R&D practices, and Logistics and Transportation practices (8%). Fluctuating supply (7%) holds the fifth position followed by equally important challenges viz., Illegal practices and lack of customers’ attitude (4% each). For the seventh position i.e. the least important among challenges it has three challenges at 3% each namely, lack of safety constraints, improper coordination and accessibility, and lack of design practices.

The results obtained for expert opinion data validation and consistency is as per the rules (Section 3.2.3; Equations (1), (2), (3)).

∑X=182.8861;CI=0.08377712032;CR=0.0537032823.

As noticed, CR is less than 0.10 and its value is 0.053 tending towards zero, thereby showcasing highly consistent opinions of experts.

### 5.2 ISM: Model and MICMAC insights

**Figure 2.**
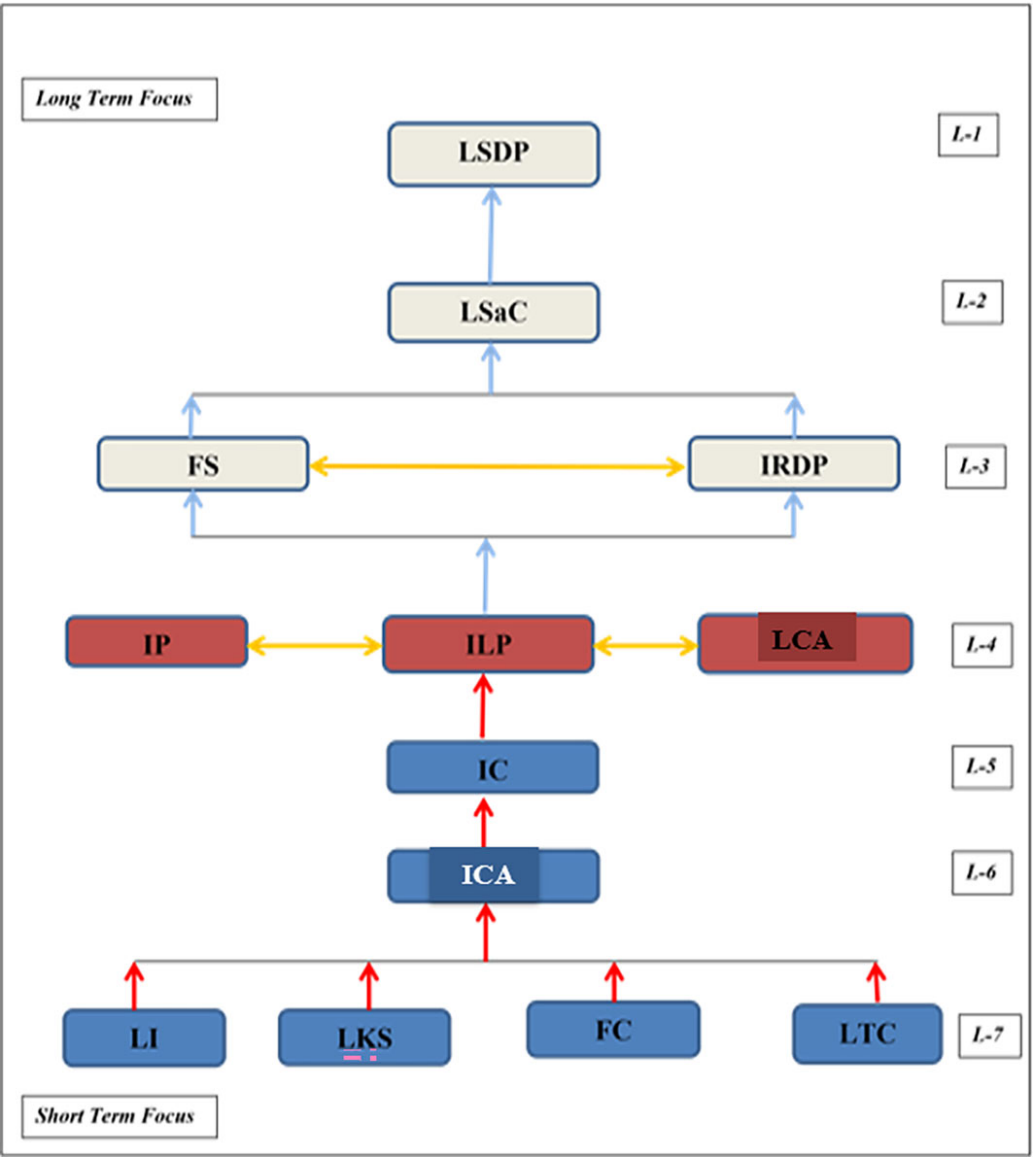
ISM model.

The ISM model elucidates very precisely the origin/present state of hindrances to e-waste mitigation and how in 7 levels reach to the cumulative hurdle of LSDP. Alternately, it can be viewed that the model lucidly explains the barriers which kindles aspirations for a revival strategy to overcome them. The ISM model delivers a structure of hindrances to e-waste implementation. The summary of the model characteristics ( Figure 2) are discussed below.

The ISM model insights can be envisioned in 7 levels with the seventh one signifying the short-term focus or the immediate problems/challenges to be addressed. These are lack of infrastructure (LI), lack of knowledge and skills (LKS), financial constraints (FC), and lack of logistics and transportation practices (LTC). Level 6 represents lack of customers’ attitude (LCA) which is triggered by hindrances of L-7. This aggravates the lack of information constraints (IC) at L-5 leading to illegal practices (IP), incoherent legal and policy constraints (ILP), and improper coordination and accessibility (ICA). These three L-4 challenges are interconnected and complement each other to build an “envelope of hindrances” encompassing the mitigation of e-waste solutions. It further becomes formidable with L-3 barriers of fluctuating supply (FS) and immature R&D practices (IRDP) which complement each other. These, in union, lead to lack of safety constraints (L-2) which finally boils down to the L-1 hindrance of lack of sustainable design practices (LSDP).

The key insights from the model can be encapsulated as follows:

• LI ( *Εε1*), LKS ( *Εε2*), FC ( *Εε3*), and LTC ( *Εε7*) are the “short-term focus” hindrances to e-waste management implementation initiation in India. They represent L-7 of the ISM model.
• The constraints that build-up due to L-7 hindrances represent ICA ( *Εε10*) {L-6} followed by IC ( *Εε4*) {L-5}.

Thus, L-7, L-6, and L-5 hindrances are the independent or driving factors to be given high priority for resolution initiation.

Hindrances *Εε8*_,_ *Εε9*, *Εε13* viz., IP, ILP, and LCA have interconnectivity creating a “sphere of hindrances” making their alleviation difficult, thus, called unstable or linkage factors.

• FS ( *Εε5*) and IRDP ( *Εε6*) hindrances are further advanced and posed in Level-3, aggravating to LSaC ( *Εε12*) and finally LSDP ( *Εε11*) representing L-2 and L-1 respectively in the ISM model. These are the dependence factors which can only be alleviated operating through L-7 to L-1, in sequence, through diligent efforts of industry personnel stakeholders/managers.

**Figure 3.**
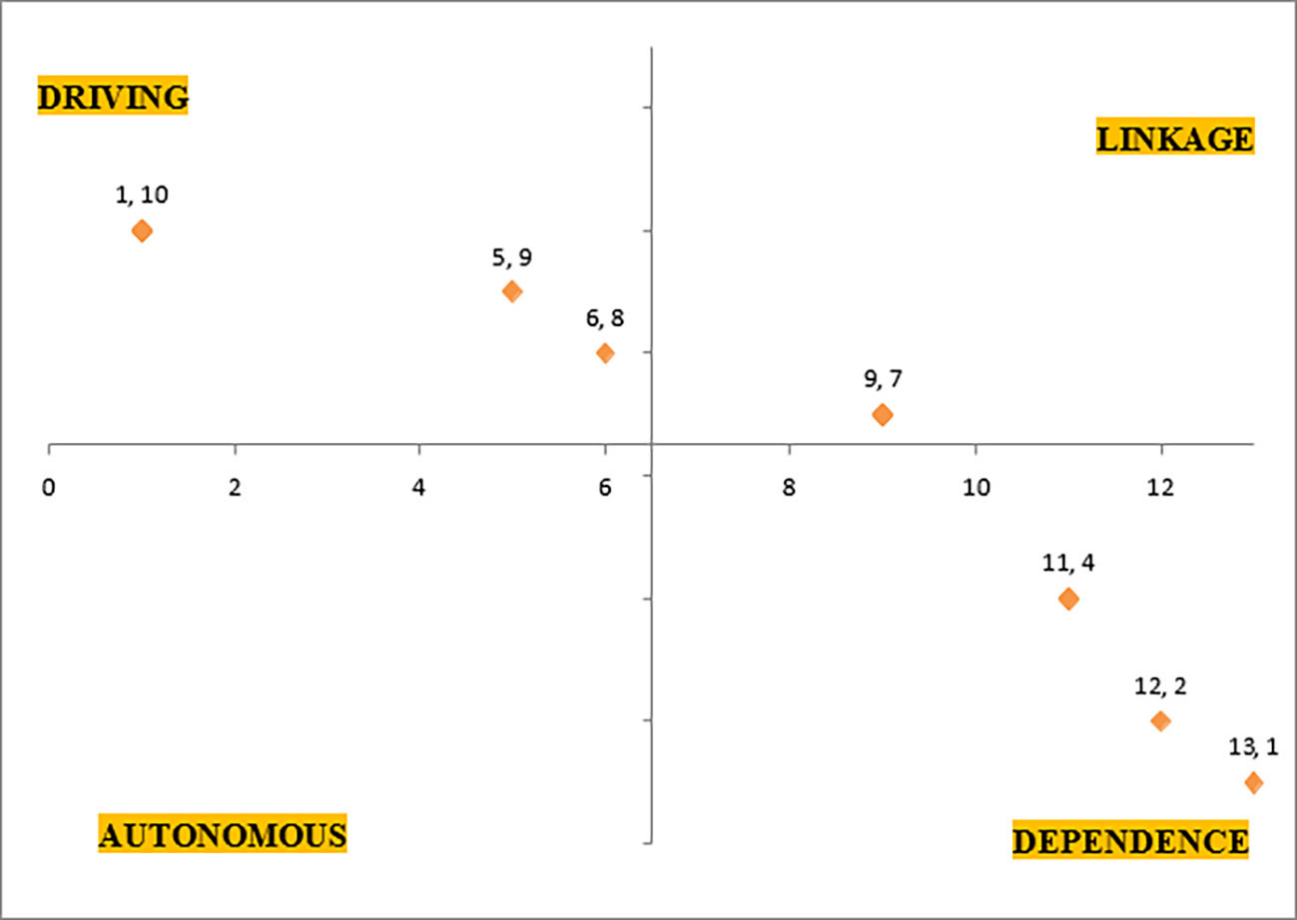
MICMAC analysis of e-waste challenges.

Figure 3 represents MICMAC analysis, which classifies the thirteen identified challenges into four sections, such as drivers, dependents, linkage and autonomous. Table 10 shows the different categories of challenges.

**Table 10.** MICMAC Coordinates and classification of challenges.

Coordinates	e-waste challenge codes	Classification
(1,10)	*Εε1, Εε2,Εε3,Εε7*	***Driving***
(5,9)	*Εε13*	***Driving***
(6,8)	*Εε4*	***Driving***
(9,7)	*Εε8, Εε9, Εε10*	***Linkage***
(11,4)	*Εε5 Εε6*	***Dependence***
(12,2)	*Εε12*	***Dependence***
(13,1)	*Εε11*	***Dependence***

## No Autonomous factors were obtained.

There is a unique observation between the results of AHP and ISM owing to the fact that the same 15 experts with their acumen provided their opinions at the same point of time. There is a horizontal trend observed that long-term focus or dependence factors are the least important hindrances, 8 out of 13 challenges of e-waste management implementation in Indian context corresponds to a trend that “the driving factors (short-term focus) to dependence factors (long-term focus) in ISM correspond to most weightage to least weightage (importance) in AHP”. Also, a narrow range between all hindrances signifies that the ISM model is fragile and susceptible to readjustments for some hindrances. Therefore, it would require proactive approach to alleviate these e-waste management implementation barriers, sooner the better.

## 6. Research implications

The implications of this research endeavor lie in demystifying the precursor challenges or hindrances to e-waste management implementation in Indian context and build awareness to the industry personnel/professionals about a structure of basic to advanced hindrances which are to be resolved in sequence as per levels through an ISM model. The authors have successfully identified 13 hindrances to initiation of e-waste management implementation in Indian context **(RQ1)** and validated them through experts’ views to find weightage of each hindrance **(RQ2)** and build a model **(RQ3)** to structure them to decode the modus operandi to alleviate them.

The key takeaways from the research are elucidated as follows:

• Hindrances identified would help stakeholders to better access the situation and to visualize e-waste mitigation initiation with a reference standard.
• The weightage of these barriers would help the industry personnel to understand the importance of each of them and better strategize their actions towards alleviating them.
• The structure provided by the ISM model through 7 levels would act as a lynchpin towards decoding the stages or order of barriers to tackle them in sequence judiciously towards a successful strategy.
• Although this research is for Indian context, it would be helpful as a base for nations newly developing e-waste management strategy or in nations who in future will take-up the e-waste mitigation agenda.
• Strong policies can be designed for which this research will act as a formidable foundation.

## 7. Conclusion

The authors, in this research, have attempted to illumine the emerging industries/organizations with intent to solve the e-waste problems in their nation through four objectives tailored to Indian context. To implement e-waste mitigation initiatives, stakeholders need to identify the potential challenges (RQ1) and gauge their importance (RQ2). The next step would be to find a structural relationship between the hindrances so that the starting point could be identified to start resolving it. RQ3 addresses this aspect through involving an ISM model to access the “structural fiber” of interrelationships in levels (1 to 7) so that it could be dealt with successfully. The research insights provided would be a guiding light to drive the vast potential of this emerging field of research.

There are certain limitations to this research are that it is a new field of research not having access to numerous experts. The observed trend between results of AHP and ISM that “hindrance weightage is inversely proportional to levels of ISM” is contextual to this research and may be explored to access its prowess globally. As an emerging field, it has a huge potential to be explored and the global threat that this e-waste issue possesses, it’s high time to treat it with extreme urgency worldwide. This research will act as a sturdy base to the emerging nations stepping into the mission of e-waste alleviation through implementing management measures.

## Ethics and consent

Ethical approval was not required for this research work.

## Data Availability

1. Figshare: SINGHDEO, ASHOK; Tripathy, Sushanta (2025). Identification of e-waste challenges.docx. figshare. Dataset. https://doi.org/10.6084/m9.figshare.29816012.v2
The project contains the following underlying data: • Table-2.docx (Identification of e-waste challenges) Data are available under the terms of the Creative Commons Attribution 4.0 International license (CC-BY 4.0). 2. Figshare: SINGHDEO, ASHOK; Tripathy, Sushanta (2025). Pairwise Comparison.docx. figshare. Dataset. https://doi.org/10.6084/m9.figshare.29816006.v2
The project contains the following underlying data: • Table-3.docx (Pairwise comparison). Data are available under the terms of the Creative Commons Attribution 4.0 International license (CC-BY 4.0). 3. Figshare: SINGHDEO, ASHOK; Tripathy, Sushanta (2025). ISM Calculations.docx. Dataset. https://doi.org/10.6084/m9.figshare.29816015.v2
The project contains the following underlying data: • Table-7.docx (ISM Calculations). Data are available under the terms of the Creative Commons Attribution 4.0 International license (CC-BY 4.0).
