# Supplementary material for: Decoding e-waste challenges with hybrid AHP and ISM Model approach: An initiative towards a cleaner future in India
Source: F1000Res. 2025 Sep 2;14:856. [Version 1] doi: 10.12688/f1000research.167041.1 (PMC12800603; doi:10.12688/f1000research.167041.1)
Supplement: Peer review history

# Reviewer report: Reviewer response for version 1

**DOI:** 10.5256/f1000research.184117.r442314

**Published:** 2026-01-13

**Authors:** Rabindra Narayan Mahapatra

**Affiliations:** 1 National Institute of Technology Agartala Department of Production Engineering, Agartala, Tripura, India

Decoding e-waste challenges with hybrid AHP and ISM Model approach: An initiative towards a cleaner future in India

 Recommendation: Accept

 It was a privilege to review the manuscript on the hot topic of e waste management globally. The manuscript addresses a highly relevant and timely issue of e-waste management particularly in the context of developing economies like India. The manuscript articulates e-waste hindrances and their prioritization through two robust MCDM techniques. The integration of ISM, MICMAC, and AHP is a strong methodological contribution. The consistency ratio in AHP confirms the reliability of expert opinions, strengthening confidence in the ranking outcomes. The availability of datasets and clear author contribution statements enhance transparency and reproducibility, which is commendable.

  The perspective put by the authors in the present manuscript showcases a paradigm shift of transforming e-waste management initiatives to the front line with an objective to “being sustainable” as the bottom line. The manuscript is novel and stands tall in timeliness and utility towards the present trends in e-waste initiatives.

 Minor Suggestions but optional

 -Briefly highlight the theoretical implications in relation to sustainability or systems-based frameworks.

 -Add a short summary of expert profiles (experience/roles) to enhance transparency.

 -Minor language and formatting polishing where required.

Is the work clearly and accurately presented and does it cite the current literature?

Yes

If applicable, is the statistical analysis and its interpretation appropriate?

Yes

Are all the source data underlying the results available to ensure full reproducibility?

Yes

Is the study design appropriate and is the work technically sound?

Yes

Are the conclusions drawn adequately supported by the results?

Yes

Are sufficient details of methods and analysis provided to allow replication by others?

Yes

Reviewer Expertise:

Supply Chain management, Manufacturing Science, Composites.

I confirm that I have read this submission and believe that I have an appropriate level of expertise to confirm that it is of an acceptable scientific standard.

# Reviewer report: Reviewer response for version 1

**DOI:** 10.5256/f1000research.184117.r442311

**Published:** 2026-01-10

**Authors:** Sourav Datta

**Affiliations:** 1 Department of Mechanical Engineering, National Institute of Technology Rourkela, Rourkela, Odisha, India

The manuscript entitled *“Decoding E-Waste Challenges with a Hybrid AHP and ISM Model Approach: An Initiative Towards a Cleaner Future in India”* addresses an important and timely problem by prioritizing the challenges associated with e-waste management. The application of a hybrid AHP–ISM approach is appropriate and methodologically sound, enabling a structured identification and hierarchical modeling of critical barriers.  Overall, the study is well organized and contributes positively to the existing literature on sustainable waste management in the Indian context. It should be **accepted with minor revision.**

 While the manuscript is well written and methodologically robust, a **minor revision could** further enhance its clarity and impact. The comments are as follows.

  

- The authors should think about giving a more thorough explanation for the criteria and specialists used for the AHP study.

 

- Will e-waste legislation change globally between 2025? If so, will they be included in Table-1?

 

- Are the results of ISM and AHP related in any way? Establishing the outcomes between two methodologies is crucial. Include this assertion in the discussion area.

 

- A more direct comparison of the results with previous research on e-waste management in India and other developing nations could improve the discussion section.
- Lastly, a few minor linguistic edits might make the text easier to understand overall.

Is the work clearly and accurately presented and does it cite the current literature?

Yes

If applicable, is the statistical analysis and its interpretation appropriate?

Yes

Are all the source data underlying the results available to ensure full reproducibility?

Yes

Is the study design appropriate and is the work technically sound?

Yes

Are the conclusions drawn adequately supported by the results?

Yes

Are sufficient details of methods and analysis provided to allow replication by others?

Yes

Reviewer Expertise:

Supply Chain Management

I confirm that I have read this submission and believe that I have an appropriate level of expertise to confirm that it is of an acceptable scientific standard.

# Reviewer report: Reviewer response for version 1

**DOI:** 10.5256/f1000research.184117.r411794

**Published:** 2025-09-24

**Authors:** PRANAV PRASHANT DAGWAR

**Affiliations:** 1 SRM University AP, Mangalagiri, Andhra Pradesh, India

**COMMENTS**

 Manuscript Number: F1000Research 2025, 14:856 - Decoding e-waste challenges with hybrid AHP and ISM Model approach: An initiative towards a cleaner future in India **.** I suggest rejection and major revision for the article.

**Comments:**

  

1. **Abstract **

- The background needs to be clearer so that it increases reader’s interest.
- Maintain same way to quote “E-waste” throughout the manuscript.
- Kindly remake the results as it is seemingly very generalised, authors needs to align it with regards to the study.
- In conclusion there is a clear use of AI – authors are requested to kindly check the manuscript for such uses and align it accordingly, Ex- “The structured model offers a foundational framework for countries—whether newly engaged or planning to implement e-waste management to adopt it as a national agenda for achieving sustainable development goals.”

 

1. **Introduction **

- In modern times, which era of modern times – please reframe from making such statements and follow other manuscript to introduce the topic in more engaging manner.
- Avoid long citations and again increase the content in introduction it is merely nothing that the authors have touched upon. 
- In paragraph two – from “in 1970s” the authors have tried covering the regulation of global nations, but Indian laws and policies are missing, authors need to rework the portion.
- The manuscript lacks novelty, and it needs a strict workup as there are many manuscripts or published articles answering the raised queries.
- The figures are not clear

 

1. **Literature Review**

- There is use of AI and it can be visible in the manuscript, authors needs to be careful with the words. Ex; Delves
- “this gigantic magnitude” please refrain from using such sentences, it questions the credibility of authors.
- Avoid use of long citations and making breaked paragraphs
- Please add figures as there is data which is very poorly explained
- Εε6: Immature R&D Practices [IRDP] – please explain immature? The manuscript, it is lacking the professional touch.

**The manuscript needs a lot of work before heading ahead -**

- The language is very poor and unprofessional
- Use of AI can be observed
- Data has not been explained properly
- Figures quality is very poor
- The technical explanation is not explained properly and very poorly attributed in manuscript.
- The novelty is lacking in the manuscript
- Please check the conclusion – with lines like “h, have attempted to illumine” the authors are not justifying the work.
- No uniformity in maintaining references – please check

Is the work clearly and accurately presented and does it cite the current literature?

Partly

If applicable, is the statistical analysis and its interpretation appropriate?

No

Are all the source data underlying the results available to ensure full reproducibility?

Partly

Is the study design appropriate and is the work technically sound?

No

Are the conclusions drawn adequately supported by the results?

No

Are sufficient details of methods and analysis provided to allow replication by others?

No

Reviewer Expertise:

E-waste management, Hazardous Waste Management, Sustainability and Circular economy, Metal Recovery, Resources recovery

I confirm that I have read this submission and believe that I have an appropriate level of expertise to state that I do not consider it to be of an acceptable scientific standard, for reasons outlined above.
